# Genome-wide transcriptome profiling and development of age prediction models in the human brain

**DOI:** 10.18632/aging.205609

**Published:** 2024-02-28

**Authors:** Joseph A. Zarrella, Amy Tsurumi

**Affiliations:** 1 Department of Health Policy and Management, Harvard T.H. Chan School of Public Health, Boston, MA 02115, USA; 2 Department of Surgery, Massachusetts General Hospital and Harvard Medical School, Boston, MA 02114, USA; 3 Shriner's Hospitals for Children-Boston, Boston, MA 02114, USA

**Keywords:** aging, machine learning, prediction model, biomarker, transcriptome

## Abstract

Aging-related transcriptome changes in various regions of the healthy human brain have been explored in previous works, however, a study to develop prediction models for age based on the expression levels of specific panels of transcripts is lacking. Moreover, studies that have assessed sexually dimorphic gene activities in the aging brain have reported discrepant results, suggesting that additional studies would be advantageous. The prefrontal cortex (PFC) region was previously shown to have a particularly large number of significant transcriptome alterations during healthy aging in a study that compared different regions in the human brain. We harmonized neuropathologically normal PFC transcriptome datasets obtained from the Gene Expression Omnibus (GEO) repository, ranging in age from 21 to 105 years, and found a large number of differentially regulated transcripts in the old and elderly, compared to young samples overall, and compared female and male-specific expression alterations. We assessed the genes that were associated with age by employing ontology, pathway, and network analyses. Furthermore, we applied various established (least absolute shrinkage and selection operator (Lasso) and Elastic Net (EN)) and recent (eXtreme Gradient Boosting (XGBoost) and Light Gradient Boosting Machine (LightGBM)) machine learning algorithms to develop accurate prediction models for chronological age and validated them. Studies to further validate these models in other large populations and molecular studies to elucidate the potential mechanisms by which the transcripts identified may be related to aging phenotypes would be advantageous.

## INTRODUCTION

It is well-established that transcriptome changes occur in various tissues throughout the course of normal aging. A study to profile gene expression changes and develop prediction models for age using transcriptome data from healthy brain samples may help elucidate the molecular changes associated with healthy aging and contribute to the prevention of age-related cognitive decline and address susceptibility to age-related neurological diseases. Such a healthy brain aging prediction model could also be useful for devising a method to assess accelerated aging, such as by applying to postmortem samples from patients with cognitive impairment or Alzheimer’s Disease, or to evaluate possible molecular mechanisms of decelerated aging, for example, in samples from centennials.

A previous study comparing transcriptome changes in the superior frontal gyrus region of the prefrontal cortex (PFC), hippocampus, and entorhinal cortex during aging, found the greatest number of differentially regulated genes with advanced age in the superior frontal gyrus [1]. Neuroimaging studies have demonstrated the significant impact of age-related changes in the activity and volume of the PFC on age-related cognitive decline (reviewed in [2]). These results suggest that using the PFC region that showed particularly significant transcriptome and physiological changes, may be effective for developing prediction models for age. Various previous studies have analyzed age-related transcriptome alterations in neuropathologically normal postmortem PFC samples [1, 3–8]. However, none of these studies specifically in the PFC have applied machine learning algorithms to identify panels of specific transcripts related to age prediction and develop models. A previous study simultaneously investigated the cortex, hippocampus, and cerebellum, rather than specifically the PFC, and applied Deep Learning Neural Networks, Support Vector Machine, and Random-Forest, RF machine learning algorithms to analyze a subset of protein-coding genes, rather than all transcripts [9].

Moreover, differences in physiological changes of the aging female versus male human brain [10] and functional connectivity differences comparing female and male PFC [11] have been reported, suggesting that molecular assessment of sexual dimorphism in the PFC during healthy aging is also important. Several previous studies aiming to characterize PFC transcriptome changes during healthy human aging did not address sex differences [3, 5, 6], or controlled for this variable with the aim to eliminate sex-dependent effects [7]. Other previous studies have aimed to elucidate gene expression differences between females and males [1, 8, 12, 13]. However, while one of them reported a larger number of differentially regulated transcripts in the male PFC than in females [1], another found the opposite result, that more transcripts were differentially regulated in the female PFC than in males [12]. A different study concluded that the female superior frontal gyrus showed accelerated aging gene expression changes that were related to Alzheimer’s Disease, compared to males [13]. Yet, in another study that profiled female versus male PFC changes in the expression of gene modules, instead of individual genes, no significant sex-specific associations were found for the modules investigated [8]. Due to these discrepancies, further studies assessing potential sexually dimorphic molecular changes of the aging healthy PFC would be advantageous.

Taken together, we aimed to profile transcriptome changes in the aging PFC overall and compare females and males, and develop prediction models for age. Machine learning algorithms are a powerful tool for developing such prediction models and can be applied to PFC transcriptome data. Performing feature selection and regularization using the least absolute shrinkage and selection operator (Lasso) [14] and Elastic Net (EN) [15] algorithms have the potential to yield accurate and interpretable prediction models for age based on linear regression. The feasibility of applying these algorithms to transcriptome data to develop prediction models has previously been explored. Furthermore, the well-established epigenetic aging clock based on DNA methylation was developed using EN [16], also showing the utility of penalized regression in developing models for age. More recently, gradient boosting-based machine learning methods, including the eXtreme Gradient Boosting (XGBoost) [17], and the Light Gradient Boosting Machine (LightGBM) [18] were developed, which also have immense potential for yielding accurate prediction models by a different approach. The SHapely Additive exPlanations (SHAP) [19, 20] algorithm was also recently developed, that can be applied to XGboost- and LightGBM-trained models to quantify SHAP scores to assess how each model feature contributes to the outcome prediction, making these complex models relatively more interpretable. However, studies applying these novel machine learning algorithms to transcriptome data in any biological context are still limited, and they also have not been previously used to develop models for healthy aging using any type of data. Therefore, comparing these different methods is expected to aid in improving prediction and to be informative to guide future studies aimed at using transcriptome information to develop prediction models, or in aging studies, more generally.

In this study, we first harmonized different postmortem neuropathologically normal PFC transcriptome datasets obtained from the Gene Expression Omnibus (GEO) repository, ranging in age from 21 to 105 to increase the sample size. Using this harmonized dataset, we identified transcripts that were differentially down- or up-regulated in middle-aged, old, and elderly PFC samples compared to young adults, and evaluated transcripts that were differentially expressed commonly in both females and males, versus unique to either. Furthermore, we applied various machine learning algorithms to develop accurate prediction models for chronological age. Our results support the notions that specific gene expression changes in the PFC are highly correlated with age, that some transcripts show female and male-specific differences, and that machine learning algorithms are useful tools for developing prediction models for age based on transcriptome information. A future large study to validate our prediction models would be advantageous, as well as molecular studies of the gene activities identified, to aid in understanding whether and how they may drive aging phenotypes or are merely passengers that alter in expression level with age.

## RESULTS

### Characterization of differentially regulated transcripts in the old and elderly samples that show strong linear correlation with age and comparisons between males and females

**Figure 1:**
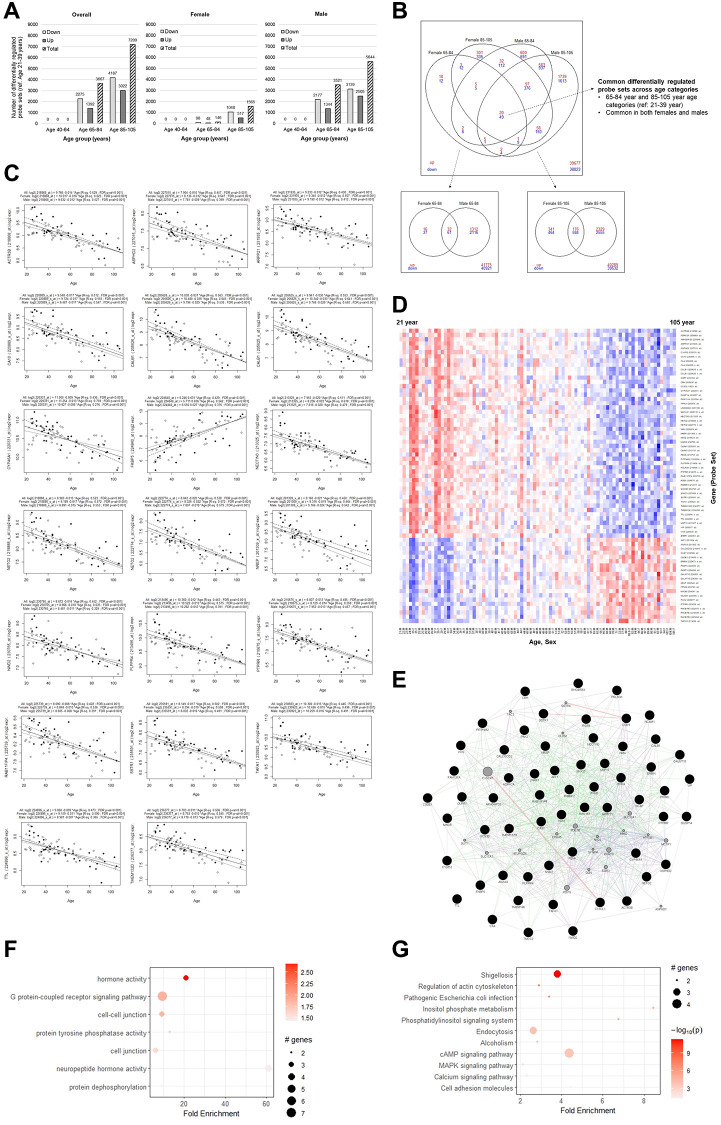
**Differential gene expression analysis overall and by sex.** (**A**) The number of probe sets with at least 1.2-fold difference and FDR-adjusted *p*-value < 0.05 to the reference young category. (**B**) Overlaps of differentially regulated transcripts between females and males for the old and elderly categories. (**C**) Univariate linear regression plots of the top 20 common differentially regulated transcripts, overall and stratified by sex. (**D**) Heatmap plot showing log_2_ expression level change of the 69 common probe sets across age. (**E**) Gene network plot, with the genes corresponding to the common transcripts represented by black filled nodes and interconnected genes in gray. Green edges indicate genetic interactions, purple edges indicate co-expression, blue edges indicate co-localization, and red edges indicate physical interactions. (**F**) GO terms (combined all, BP, MF, CC), and (**G**) KEGG pathway enrichment analysis.

Using data obtained from Gene Expression Omnibus (GEO), we analyzed the transcriptome of postmortem PFC samples from neuropathologically normal subjects, ranging in age from 21 to 105 (Supplementary Table 1). We identified probe sets showing significant change (applying a cut-off of ≥1.2-fold change and FDR *p*-value < 0.05), comparing young adult (21–39 years) samples as the baseline, to middle age (40–64 years), old (65–84 years) and elderly (85–105 years) samples (Figure 1A, Supplementary Table 2). We identified overlaps across age categories within each sex (Supplementary Table 3) and overlaps among the sexes (Supplementary Table 4). Overall, we found more downregulated probe sets compared to upregulated probe sets, and as expected, the number of differentially regulated probe sets increased with the older age category. We did not find significant differentially regulated genes (DEGs) in middle-aged (40–64 years) samples compared to the reference young samples, which may be due to the small sample size of our study. For the old (65–84 years) category, we found 3,667 probe sets to be differentially regulated (2,275 downregulated and 1,392 upregulated), and this increased to 7,209 (4,187 downregulated and 3,022 upregulated) in the elderly (85–105 years) category combining females and males (Figure 1A, Supplementary Table 2). In both the age categories, more differentially regulated probe sets were found among males (3,521 total with 2,177 downregulated and 1,344 upregulated in the old, and 5,644 total with 3,139 downregulated and 2,505 upregulated in the elderly), compared to females (146 total with 98 downregulated and 48 upregulated in the old, and 1,565 total with 1,048 downregulated and 517 upregulated in the elderly). This may be due to the sample size of females being smaller overall (34 female vs. 53 male). However, while there were fewer female samples in the old group (5 female vs. 10 male), there were more in the elderly group (7 female vs. 5 male), and the differences in DEGs may still be informative (Supplementary Table 1).

We identified 69 probe sets that were common DEGs in both old and elderly females and males, which included 49 downregulated probe sets, mapping to 44 genes, and 20 downregulated probe sets corresponding to 16 genes (Figure 1B, Supplementary Table 5). To identify transcripts that continually changed with age, we also performed univariate linear regression with all the probe sets to identify transcripts whose log_2_ expression had a notable linear relationship with age (Supplementary Table 6). All 69 overlapping differentially regulated transcripts also showed a strong linear relationship with age (coefficient estimates’ FDR-adjusted *p*-value < 0.05). Among these probe sets, the goodness-of-fit R-squared (R^2^) was ≥0.4 for 26 of them, ≥0.3 for 60 of them, and ≥0.2 for all 69 of them, suggesting that they likely represent continuous age-related gradual gene expression changes (Supplementary Table 6). The linear regression plot of the top 20 representative probe sets (Figure 1C), and the heatmap plot of the 69 probe sets ordered across age (Figure 1D), also show that they change in expression gradually with age.

Among the downregulated genes, two probe sets were each found for *Carbonic Anhydrase 4* (*CA4*), *Calbindin 1* (*CALB1*), *Neuropilin and Tolloid Like 2* (*NETO2*), and *Olfactomedin1* (*OLFM1)*. Among the upregulated genes, there were three probe sets mapping to *Rho Related BRB Domain Containing 3* (*RHOBTB3*), and two probe sets each for *FKBP prolyl isomerase 5* (*FKBP5*) and *Polypeptide N-Acetylgalactosaminyltransferase 15* (*GALNT15*). Having found multiple probe sets for the same genes suggest that they are likely important. Two *Transmembrane Protein* (*TMEM*) family members, *TMEM132D* and *TMEM196* were also found among the downregulated probe sets. Multiple genes related to the G-protein coupled receptor pathway, *Adrenoceptor Alpha 2A* (*ADRA2A*), *C-X3-C Motif Chemokine Ligand 1* (*CX3CL1*), *Neuromedin U* (*NMU*), *Phospholipid Phosphatase Related 4* (*PLPPR4*), *Regulator of G Protein Signaling 8* (*RGS8*), and *Vasoactive Intestinal Peptide* (*VIP*) were also found.

The gene network plot found that the overall DEGs were highly interconnected (Figure 1E). Gene Ontology (GO) enrichment analysis found that the DEG set was associated with GO terms that were mainly related to protein post-translational modifications, cell junctions, hormone activity, and G-protein coupled receptor signaling pathway (Figure 1F). Kyoto Encyclopedia of Genes and Genomes (KEGG) pathway terms implicated metabolism, cytoskeleton, cell adhesion, alcoholism, various signaling pathways, and infection responses (Figure 1G), which have previously been implicated in aging.

### Distinct female and male-specific PFC transcripts were identified

**Figure 2:**
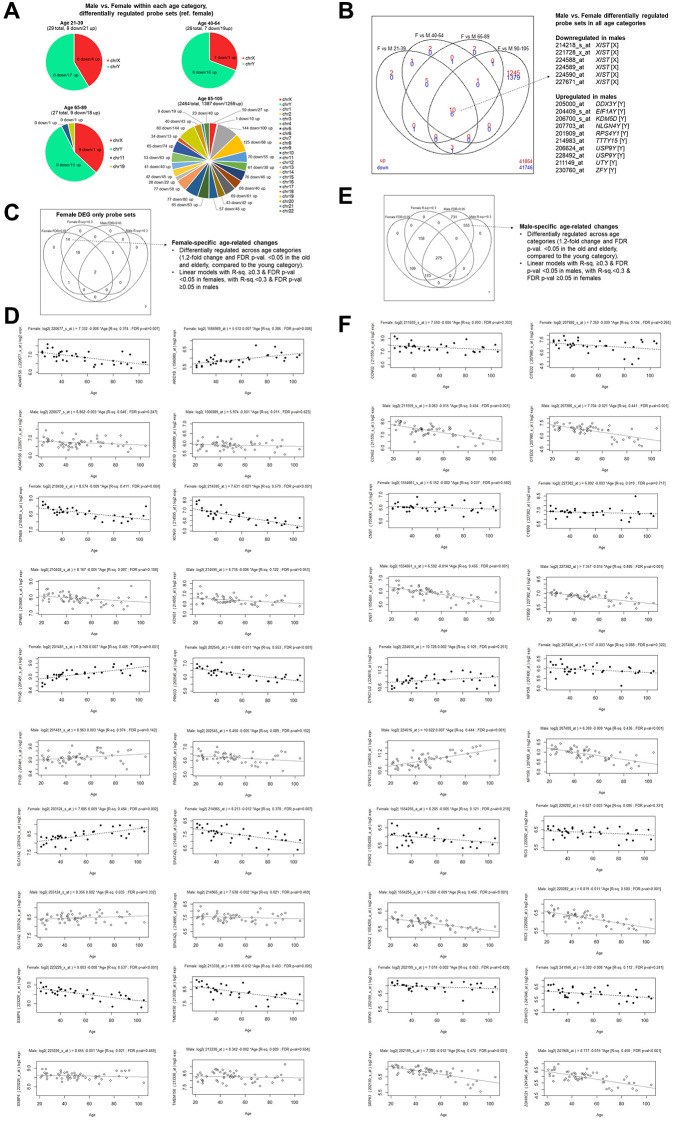
**Female versus male gene expression analyses within age categories.** (**A**) Chromosomal locations of probe sets found to have at least 1.2-fold difference and with FDR-adjusted *p*-value < 0.05 comparing females and males within each age category. (**B**) Overlaps of transcripts found to be differentially expressed in females and males (reference: females) in different age categories. The 16 probe sets found in all age categories are listed, with their probe set annotation, gene name, and chromosomal location indicated in square brackets. (**C**) Female-specific age-related transcripts by the stringent criteria. Transcripts that were found to be differentially regulated in the old and elderly categories compared to the young (at least 1.2-fold difference and FDR *p*-value < 0.05) only in females (35 probe sets) were assessed whether they were also associated with age in a univariate linear regression model (R^2^ value ≥0.3 and coefficient estimate *t*-test *p*-value < 0.05) only in females. (**D**) Linear regression models were plotted by sex, where the top panels with black circles correspond to females and the bottom panels with open circles correspond to males. Top ten representative probe sets with high R^2^ and FDR *p*-value < 0.05 only among females, while having higher FDR *p*-value among males, were plotted. (**E**) Male-specific age-related transcripts by the stringent criteria. Transcripts that were found to be differentially regulated in the old and elderly categories compared to the young (at least 1.2-fold difference and FDR *p*-value < 0.05) only in males (1999 probe sets) were assessed whether they were also associated with age in a univariate linear regression model (R^2^ value ≥0.3 and coefficient estimate *p*-value < 0.05) only in males. (**F**) Linear regression models were plotted by sex, where the top panels with black circles correspond to females and the bottom panels with open circles correspond to males. Top ten representative probe sets with high R^2^ and FDR *p*-value < 0.05 only among males, while having higher FDR *p*-value among females, were plotted.

We also compared transcriptome differences between females and males within each age category, rather than comparing differentially regulated probe sets relative to the reference young category (Figure 2A, 2B, Supplementary Table 7). There were 29 differentially regulated female versus male differentially regulated probe sets within the young group, and 26 differentially regulated probe sets within the middle-aged group, both of which included only genes encoded on sex chromosomes. For the old age group, most of the 27 differentially regulated probe sets were found on sex chromosomes, although some were also two found on autosomes. On the other hand, for the elderly group, the number of differentially regulated probe sets between females and males increased notably and was found across all the different chromosomes, which may suggest that more differences arise in later years.

As expected, most of the probe sets found to be downregulated among males compared to females were mapped to the X-chromosome, and on the other hand, most probe sets found in the upregulated sets were mapped to the Y-chromosome (Figure 2A). Exceptions to this trend included pseudoautosomal regions and known to escape X-chromosome inactivation, such as *acetylserotonin O-methyltransferase-like* (*ASMTL*) and *CD99 Molecule* (CD99), *colony-stimulating factor 2 receptor, alpha* (*CSF2RA*), and *GTP binding protein 6 (putative)* (*GTPBP6*) [21], among others. Six probe sets corresponding to the well-established female-specific transcript, *X inactive specific transcript* (*Xist*), were significantly downregulated in males in all four age categories, as expected. Moreover, ten transcripts found to be upregulated in males in all four age categories were all encoded on the Y-chromosome, as expected, including *DEAD-Box Helicase 3 Y-linked* (*DDX3y*), *Eukaryotic Translation Initiation Factor 1A Y-linked* (*EIF1AY*), *Ribosomal Protein S4 Y-Linked 1* (*RPS4Y1*), *Ubiquitin Specific Peptidase 9 Y-linked* (*USP9Y*) (Figure 2B). These transcripts, therefore, do not appear to be age-related sexual dimorphic gene expression, however, provide further support for the results.

To identify transcripts showing gradual down- or up-regulation with age only in females or only in males, we used stringent criteria of both evidence from DEG (≥1.2-fold change and FDR *p*-value < 0.05 in both the old and elderly compared to the young only among females or among males), and linear regression analyses (R^2^ ≥0.3 and FDR *p*-value < 0.05 with age). Among female old and elderly overlapping DEGs, we found 14 probe sets that also had a significant linear relationship with age (10 declining, and 4 increasing probe sets) (Figure 2C, 2D, Supplementary Table 6). We also used the same stringent criteria among males to identify 555 probe sets (385 declining and 170 increasing probe sets) (Figure 2E, 2F, Supplementary Table 6).

### Applying four different machine learning algorithms yielded highly accurate age prediction models

**Figure 3:**
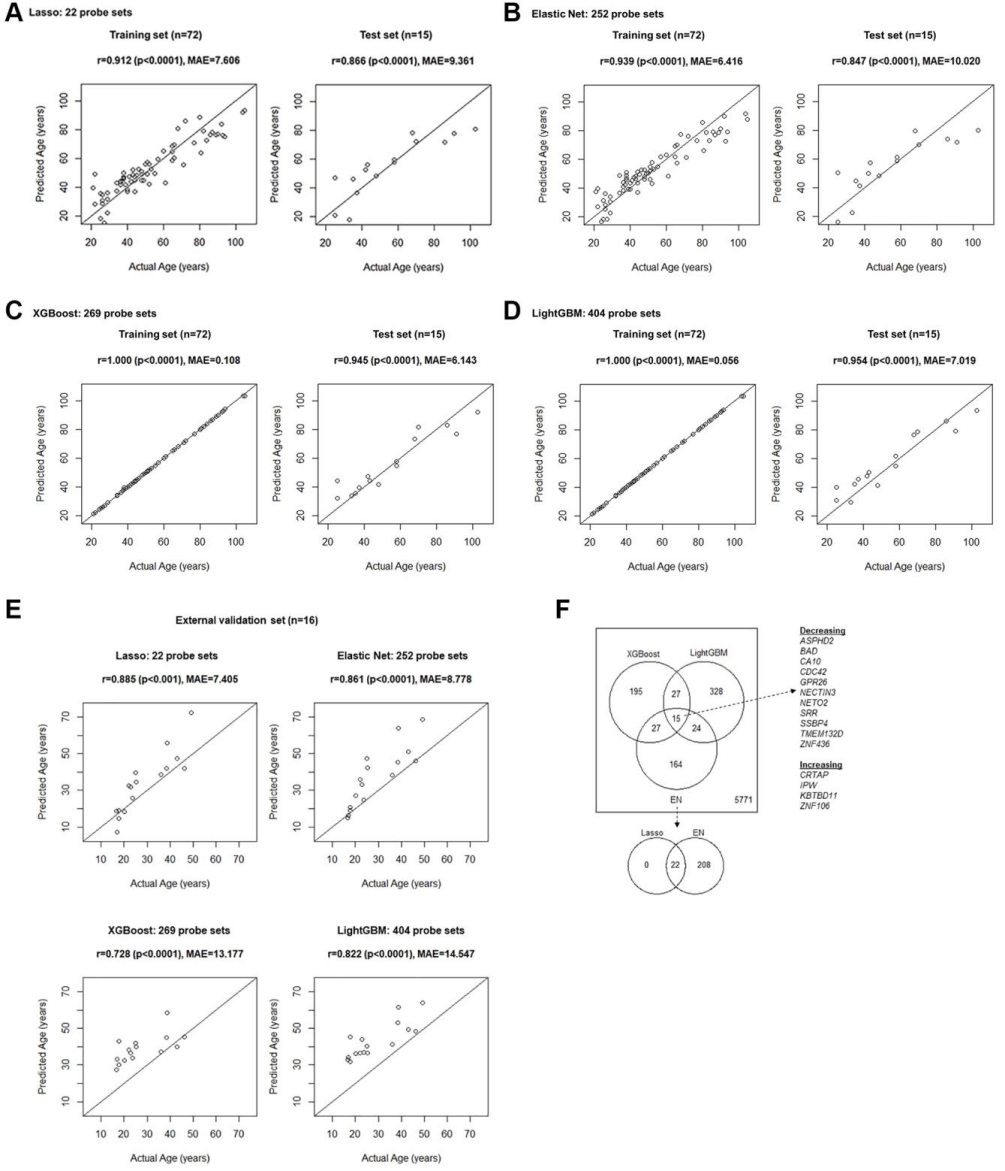
Age prediction model fit results in the training set and validation in the test set, developed with (**A**) Lasso, (**B**) EN, (**C**) XGBoost, and (**D**) LightGBM machine learning algorithms. (**E**) Results of the prediction by applying models developed using the training set to the external validation set. (**F**) Overlaps of genes whose expression levels were found to be important for the age prediction. Initially, 6,551 unique genes were considered, corresponding to the 9,296 probe sets showing linear relationship with age (FDR *p*-value < 0.05) in univariate linear regression modeling.

We applied well-established algorithms based on penalized regression methods, Lasso and EN, as well as more recently developed gradient boosting-based algorithms, XGBoost and LightGBM to the harmonized genome-wide transcriptome dataset to develop prediction models for age. The Lasso model included 22 probe sets (each corresponding to a unique gene) and yielded accurate age prediction in the training set (r = 0.912 and MAE = 7.606 compared to the actual age) and was validated in the test set (r = 0.866 and MAE = 9.361) (Figure 3A, Supplementary Table 8). The EN model, included 252 probe sets (corresponding to 230 unique genes), and also yielded accurate age prediction in the training set (r = 0.939 and MAE = 6.416) and was validated in the test set (r = 0.847 and MAE = 10.020) (Figure 3B, Supplementary Table 8). The XGBoost model yielded 269 probe sets (corresponding to 264 unique genes) with mean SHAP scores showing importance for prediction. This model had exceptional accuracy in the training set (r = 1.000 and MAE = 0.108), and validated in the test set (r = 0.945 and MAE = 6.143) (Figure 3C, Supplementary Table 8). The LightGBM model yielded 404 probe sets (corresponding to 394 unique genes) with importance for prediction, also resulted in exceptional prediction in the training set (r = 1.000 and MAE = 0.056) and validated in the test set (r = 0.954 and MAE 7.019) (Figure 3D, Supplementary Table 8). To further ensure that the predictors and the model we developed are indeed generalizable, we additionally applied each of them to an external validation set (Figure 3E). The Lasso (r = 0.885 and MAE = 7.405) and EN (r = 0.861, MAE = 8.778) models performed slightly better than the XGBoost (r = 0.728 and MAE = 13.177) or LightGBM (r = 0.822 and MAE = 14.547) models, suggesting that the latter models may be more overfit. The latter models tended to provide an overestimated prediction.

We evaluated overlaps in unique genes whose transcript levels were found to be important for age prediction by the EN, XGBoost, and LightGBM models (Figure 3F), which may strongly suggest that they are indeed impactful. All the probe sets from the Lasso model were included in the EN model and thus the overlaps among the three models, EN, XGBoost and LightGBM were evaluated. We found 15 genes including *Aspartate Beta-Hydroxylase Domain Containing 2* (*ASPHD2*), *BCL2 Associated Agonist of Cell Death* (*BAD*), *Carbonic Anhydrase 10* (CA10), *Cell Division Cycle 42* (*CDC42*), *G Protein-Coupled Receptor 26* (*GPR26*), *Nectin Cell Adhesion Molecule 3* (*NECTIN3*), *Neuropilin And Tolloid Like 2* (*NETO2*), *Serine Racemase* (*SRR*), *Single Stranded DNA Binding Protein 4* (*SSBP4*), *Transmembrane Protein 132D* (*TMEM132D*), and *Zinc Finger Protein 436* (*ZNF436*), whose transcript levels were found to decrease linearly with age, and *Cartilage Associated Protein* (*CRTAP*), *Imprinted In Prader-Willi Syndrome* (*IPW*), *Kelch Repeat and BTB Domain Containing 11* (*KBTBD11*), and *Zinc Finger Protein 106* (*ZNF106*) found to increase with age (Figure 3F).

## DISCUSSION

The goal of our study was to profile PFC transcriptome changes during healthy human aging overall and comparing potential differences between female and male samples, as well as developing chronological age prediction models by various methods. As expected, we found more DEGs with increasing age category. Furthermore, we used stringent criteria of significant differential expression across old and elderly categories, together with evidence of strong linear association showing gradual age-related decline or increase across age, in order to identify age-related transcripts in the overall population. Multiple probe sets were found for genes previously implicated in aging or aging-related diseases, including downregulated genes *CA4, CALB1, NETO2*, and *OLFM1*, and upregulated genes, *FKBP5, RHOBTB3*, and *GALNT15*, suggesting that they are indeed age-related transcripts and validate our approach. We also found various transcripts that were found to be important for age prediction commonly by the different models constructed (*ASPHD2, BAD, CA10, CDC42, GPR26, NECTIN3, NETO2, SRR, TMEM132D*, and *ZNF436* decreasing transcripts, and *CRTAP, IPW, KBTBD11*, and *ZNF106* increasing transcripts), indicating their significance. Our approach identified genes that were previously implicated in aging, as well as new ones that may warrant further investigation.

We found evidence of *CA4* downregulation with age and importance for prediction. Carbonic anhydrases (CA) catalyze the conversion of carbon dioxide to bicarbonate and important for pH regulation, and are expressed in glial cells in the brain, as well as widely in other tissues (reviewed in [22]). *CA4* mutations have been found to be associated with retinal diseases and photoreceptor degeneration [23–25], and also found to be downregulated in various tumors [26–29]. The downregulation of these *CA4* detected in this study may thus suggest deregulation of carbon dioxide and pH homeostasis in the aging brain. *CA10*, encoding a non-catalytic related protein acting as a ligand for neurexins in neurons [30], was also found in our study as a transcript important for age prediction. It is also downregulated in gliomas, with lower expression among glioma patients associated with high grade subtype and shorter survival [31]. Considering its important role in synaptic Ca^2+^ signaling via neurexin, as well as its association with glioma, an aging-related disease, our results showing the importance of *CA10* transcript in age prediction may be important.

Consistent with previous findings, we detected downregulation of multiple *CALB1* probe sets with age [5, 32]. *CALB1*, a calcium-binding protein that buffers intracellular Ca^2+^ to maintain signaling homeostasis, has been demonstrated to be important for memory [33], and its overexpression has been shown to have neuroprotective effects in a murine Parkinson’s disease (PD) model [34]. It was found to be downregulated in Huntington’s disease (HD) postmortem PFC samples [35] and in a murine HD model [36]. Reduced CALB1-expressing neurons have been associated with cell death and damaged sites in HD [37]. In a mouse Alzheimer’s disease (AD) model, ablation of *CALB1* was found to exacerbate pathogenesis [38], and in both healthy and AD human patient tissues, its downregulation was found to be correlated with that of *chitotriosidase* (*CHIT1*) that is involved with AD pathogenesis [39], demonstrating its protective role against neurodegenerative diseases. Thus, it may be plausible to speculate that decreased *CALB1* expression during aging and altered Ca^2+^ signaling could result in increased risk of various neurodegenerative diseases.

*NETO2* was also found among genes with multiple DEG probe sets and found to be important for age prediction, highlighting its importance. *NETO2* and *Somatostatin receptor 1* (*SSTR1*), which was also a DEG identified in our study, has previously been found to be downregulated in post-mortem aging and AD hippocampus [40], as well as the aging brain in rats [41]. The crucial function of NETO2 in the central nervous system is well-established, as an auxiliary protein for the kainate [42–44] and NMDA [45] glutamate receptors. It also interacts with neuron-specific K+/Cl− cotransporter type 2 (KCC2) to regulate neuronal chloride homeostasis and GABAergic inhibition [46]. Furthermore, it has been reported that *NETO2* is aberrantly expressed in various cancers, and its knockdown in human colorectal carcinoma cell line led to changes in the expression of transcripts involved in the circadian rhythm and various major signaling pathways, including Wnt, transforming growth factor (TGF)-β, Janus kinase (JAK)-signal transducer and activator of transcription (STAT), mitogen-activated protein kinase (MAPK), and phosphatidylinositol 3-kinase (PI3K)/protein kinase B (AKT) pathways [47].

*OLFM1* was also found to be downregulated with age in our study, validating previous findings [48, 49]. Age-related decline in the olfactory system is well documented (reviewed in [50]), and the disruption of the *OLFM1* gene in mice has been shown to result in brain dystrophy and behavioral abnormalities [51]. *ACTR3B* downregulation in the aging brain has also been reported previously [8]. *Transmembrane Protein* (*TMEM*) family members, *TMEM132D* and *TMEM196* were also found among the stringent downregulated transcripts, and *TMEM132D* was also found to be important among the different prediction models. Polymorphisms of the *TMEM* family of genes have been shown to be associated with Parkinson’s disease [52, 53]. *KBTBD11* polymorphism was also found to be associated with cognitive decline [54]. Multiple transcripts related to the G-protein coupled receptor pathway (*ADRA2A*, *CX3CL1*, *NMU*, *PLPPR4*, *RGS8*, and *VIP* as DEGs, and *GPR26* as a common predictor of the different models) were also found in our study, which have been previously linked with the aging brain (reviewed in [55]). These results support the validity of our approach.

*NECTIN3*, another gene found to be downregulated and important for prediction, has been shown to be crucial for hippocampus-dependent memory [56], and documented to become reduced in the hippocampus upon stress [57, 58]. *CDC42* has been shown to be important for synaptic plasticity and memory recall [59] and contribute to hematopoietic stem cell aging [60]. *SRR* downregulation in the aging rat hippocampus has been shown to play a role in cognitive decline [61]. Understanding the contribution of the regulation of these genes in the human PFC and aging phenotypes may be beneficial.

*FKBP5* was found to be upregulated in our study, which was previously shown to be demethylated and upregulated in peripheral blood with aging and stress [62], and similarly in mice, it was shown to be demethylated with age [63], and its role in age- and stress-related inflammation was demonstrated. Another upregulated gene, *GALNT15* has been previously shown to be upregulated in low grade glioma [64]. *RHOBTB3*, another transcript found to be upregulated, has been associated with AD in a genome-wide single nucleotide association study [65]. Other upregulated genes identified included *MOAB* and *GFAP*, which were also previously shown in the aging human brain [5], validating our findings. Taken together, these upregulated transcripts found in our study that have been linked to aging-related diseases in the brain, but not yet with expression changes in the healthy aging may warrant further investigation. Furthermore, additional studies on the link between human brain aging or aging-related cognitive decline and changes in the expression of *ASPHD2, BAD, CRTAP, IPW*, and *ZNF106* which remain elusive, may also be advantageous. Taken together, future investigations of these newly identified biomarkers of aging may yield additional informative results.

When we assessed sex differences, we found notably more DEGs in males compared to females overall, which is similar to a previous finding by Berchtold et al. [1] and opposite to the result found by Wruck et al. [12]. One disadvantage of our study is that the sample size was overall smaller for the females compared to the males, however, there were relatively more female samples in the elderly category, and given the substantially larger number of differentially regulated transcripts found in males in both the old and elderly categories, these results may still be important. For example, in our study, we found four probe sets mapping to genes implicated in neurological diseases such as AD, including *Homeodomain Interacting Protein Kinase 2* (*HIPK2*) [66] and *platelet-activating factor acetylhydrolase 1b* (*PAFAH1B1*) [67], and three probe sets mapping to genes including *BIN1* [68, 69], *APLP2* [70, 71], and *VCAN* [72], more specifically in males. However, this contrasts with epidemiological findings describing disproportionately more prevalent cases of AD and dementia among females [23]. Additional molecular mechanistic studies to understand how dimorphic expression changes occur and their effects would be informative. In support of our finding detecting molecular differences between female and male PFC, a recent study reported a lower metabolic brain age compared to chronological age in old female human subjects compared to male [73]. On the other hand, a recent study concluded that when controlling for overall size, sex differences in structure, connectome, and processing could not be found [74]. Therefore, further work on evaluating sexually dimorphic changes in various molecular markers of age and comparing them with chronological age would be informative.

When comparing female and male samples within each age category, rather than differentially regulated transcripts relative to the young, we found, for the most part, X- and Y-chromosome encoded transcripts. These included multiple probe sets for *Xist* in the male-downregulated set, as expected, and genes encoded on the Y-chromosome (*DDX3y, EIF1AY, KDM5D, NLGN4Y1, TTTY15, USP9Y, UTY, ZFY*) in the male-upregulated set, providing added evidence of the validity of our analysis results. The elderly category had the largest number of DEGs comparing females and males, and spread across autosomes as well as the sex chromosomes. This increase in both the number of DEGs and different chromosomal locations may imply that gene expression patterns between females and males become more divergent with age.

Among our main goals was to develop prediction models for age by employing well-established methods, Lasso and EN, based on regularized regression, and novel methods, XGBoost and LightGBM, based on gradient boosting. Making these comparisons are informative, as applications of XGBoost and LightGBM to transcriptome data are still limited and they have also not been utilized yet in aging studies. We harmonized various studies to achieve a sample size that made it possible to perform model building, although it was smaller than ideal. Despite the small sample size of our study, accurate age prediction models were developed, and found to be generalizable by applying them to a test set and an external validation set. The different model-developing techniques resulted in largely different transcripts being selected as important for prediction. This finding is informative and suggests that employing different algorithms could aid in improving the discovery of predictors of outcomes in future studies. Our results showed that gradient boosting machine-based methods notably improved prediction in the training set compared to regularized regression-based methods, however, did not improve prediction in the external validation set. A recent study directly comparing diabetes outcome prediction models built by Lasso, XGBoost, and LightGBM using clinical variables, rather than transcriptome data, also found the overall performance of the different models to be comparable [75]. Therefore, the authors concluded that established regression-based models may still be advantageous, given that they are interpretable and more easily implemented in practice.

Additional studies to externally validate our findings in a larger study would be highly informative and would provide a more concrete assessment of which model’s performance is best. In addition to validation in larger studies, mechanistic studies would also be advantageous for further elucidating potential biological roles and validating the age-related and sexually dimorphic transcripts identified in our study. Moreover, this study was limited to comparing samples labeled as female or male, presumably by assignment at birth, and more inclusive studies, which are currently lacking, would be important.

## METHODS

### Data availability and study population

The datasets used in this study were obtained from the Gene Expression Omnibus (GEO) repository (http://www.ncbi.nlm.nih.gov/geo/) [76]. The three data series all used microarray transcriptome, Affymetrix Human Genome U133 Plus 2.0 Arrays. Data series GSE53890 included postmortem PFC samples from 41 neuropathologically normal subjects, 24 to 106 years old, with 21 labeled as females and 20 as males [7]. GSE21138 included postmortem PFC (Brodman Area 46 (BA46)) from schizophrenic and matched control samples [77], from which 29 control arrays were used, with age range 21 to 80 years old, with 5 labeled as females and 24 as males. GSE53987 was derived from a study that profiled the PFC, striatum, and hippocampus of subjects diagnosed with schizophrenia, bipolar, or major depressive disorder, and matched controls [78], from which, the 19 control PFC arrays aged 22 to 68 years, with 9 labeled as females and 10 males, were included in this study.

### Microarray dataset pre-processing and harmonization

First, the three data series [7, 77, 78] were accessed from GEO using the “GEOquery” package [79]. The CEL files obtained were normalized and converted to log_2_ expression data with frozen robust multi-array average (fRMA) using the “frma” package [80]. Then the original 54,675 probe sets were filtered to keep only those with Entrez Gene ID annotation using the “genefilter” package [81], to yield 43,135 probe sets for subsequent analyses. The “limma” package [82] was applied to account for batch effect and for implementing quantile normalization between arrays and visually inspected with a box plot. Principal components analysis (PCA) was performed using the base “stats” package [83], where two samples were identified as outliers and removed from the study. The final harmonized dataset included 87 samples, ranging in age from 21 to 105, with 34 labeled as females and 53 as males. The PCA results of the final harmonized dataset were visualized with the “factoextra” package [84] (Supplementary Figure 1). The GSM number for each CEL file used in this study and corresponding age and sex are provided in Supplementary Table 1.

### Differentially expressed gene (DEG) analysis

The data from the 87 subjects were categorized into age groups of 21–39-year-old, 40–64-year-old, 65–84-year-old, and 85–105-year-old. The “limma” package [82] was used to identify probe sets with at least a 1.2-fold change compared to the reference 21–39-year-old category and to compute the Benjamini-Hochberg false discovery rate (FDR)-adjusted *p*-values. Those showing overlap across all age categories in all subjects, versus only in female subjects, versus only in male subjects were identified and plotted in a Venn diagram. To compare the fold change between sexes between each age category, the female group was used as the reference.

### Univariate linear regression models

Univariate linear regression models were constructed for age versus each of the 43,135 probe sets’ log_2_ expression values in the base “stats” package [83]. Coefficient estimate FDR-adjusted *p*-value < 0.05 was determined to assess significant linear relationship with age.

### Gene ontology (GO) analysis

Functional annotation analysis was conducted to understand biological mechanisms related to the 69 DEG probe sets, using the “pathfindR” package [85]. The fold enrichment and FDR-adjusted *p*-values were obtained for all the Gene Ontology (GO) terms combined (Biological Process (BP), Molecular Function (MF), and Cellular Component (CC)), and for the Kyoto Encyclopedia of Genes and Genomes (KEGG) pathway terms. For both enrichment analyses, the Biological General Repository for Interaction Datasets (BioGRID) and STRING database were used for the Protein-Protein Interaction (PPI) Network search. Significant terms (FDR *p*-value < 0.05) that also had at least two genes identified in the term, were plotted in a bubble chart.

### Heatmap and network plots

The heatmap of the 69 DEG probe sets was created using the “gplots” package [86]. The network plot was constructed using the GENEMania Cytoscape plug-in [87].

### Machine learning application to develop age prediction models

#### Data description

Among the 87 subjects, 72 (~80%) were randomly selected as the training set and the other 15 (~20%) were used as the test set, using the “caret” package [88]. The age and sex distributions of the training versus the test set were evaluated to ensure that they were comparable and similar to the overall distribution (Supplementary Table 9). The machine learning algorithms below were performed using expression levels of 9,296 probe sets (mapping to 6,551 unique genes) with coefficient estimate FDR *p*-value < 0.05 when performing univariate linear regression indicating that they are associated with age overall.

### Least absolute shrinkage and selection operator (Lasso) and elastic net (EN)

We employed the Lasso and EN regression using the “glmnet” package [89], to select features. First, a five-fold CV was performed to find the regularization parameters based on obtaining the minimum mean square error (MSE) (Supplementary Table 10). Using these parameters, a stringent panel of 22 probe sets was selected with Lasso, and a more comprehensive panel of 252 probe sets was selected with EN. Then, ridge regression was performed on the log_2_ expression levels of the selected probe sets using the training set, based on λ giving minimum MSE with leave-one-out cross-validation (LOOCV), to yield the final model’s coefficient estimates.

### eXtreme gradient boosting (XGBoost) light gradient boosting machine (LightGBM)

XGBoost was applied using the “xgboost” package [90], and LightGBM was applied using the “lightgbm” package [91]. Five-fold CV was performed to find the parameters that minimized the root MSE (Supplementary Table 10), and the final models were constructed using the training set. For both these models, the SHapely Additive exPlanations (SHAP) score was found for each of the probe set expression levels with the “SHAPforXGBoost” package [92]. The XGBoost model included 269 probe sets and the LightGBM model included 404 probe sets that had mean feature importance and SHAP value of above 0.

### Test set validation

Each of the four final models, developed in the training set, was applied to the test set to find the predicted ages, and the concordance between the predicted versus the actual ages was compared with Pearson’s correlation and finding the mean absolute error (MAE).

### External validation

Using the “GEOquery” package [79] to access the GEO repository, adult postmortem PFC transcriptome CEL files generated by the Affymetrix Human Genome U133 Plus 2.0 Array in data series GSE13564 were obtained [4]. Similarly to the training set, they were converted to log_2_ expression data using the “frma” package [80], and filtered using the “genefilter” package to keep only those with Entrez Gene ID annotation [81]. The “limma” package [82] was used to perform quantile normalization, considering the previously harmonized training dataset as the target quantile, as well as to remove batch effect. Each of the four final models was applied to this external validation set to find the predicted ages, and the concordance between the predicted versus the actual ages was compared with Pearson’s correlation and finding the MAE.

### Dataset and code availability

R version 4.1.3 [83] was used. The harmonized training and CV sets, external validation set, and R code scripts are deposited in Github (https://github.com/feimae/PrefrontalCortextAgingModel).

## Supplementary Materials

Supplementary Figure 1

Supplementary Table 1

Supplementary Table 2

Supplementary Table 3

Supplementary Table 4

Supplementary Table 5

Supplementary Table 6

Supplementary Table 7

Supplementary Table 8

Supplementary Tables 9 and 10
