# Supplementary figures and images for: Genome-wide transcriptome profiling and development of age prediction models in the human brain
Source: Aging (Albany NY). 2024 Feb 28;16(5):4075–94. doi: 10.18632/aging.205609 (PMC10968712; doi:10.18632/aging.205609)

[www.aging-us.com](http://www.aging-us.com)

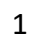

Supplement: Supplementary Figure 1 [file aging-16-205609-s001.pdf]
