# Supplementary material for: Genome-wide transcriptome profiling and development of age prediction models in the human brain
Source: Aging (Albany NY). 2024 Feb 28;16(5):4075–94. doi: 10.18632/aging.205609 (PMC10968712; doi:10.18632/aging.205609)
Supplement: Supplementary Tables 9 and 10 [file aging-16-205609-s010.pdf]

SUPPLEMENTARY TABLES

Supplementary Table 9. The age and sex distributions of the training and test set.

	Total (*N* = 87)	Training Set (*N* = 72)	test set (*N* = 15)	*p*-value (Training vs. test set)
Age: mean (min–max)	52.8 (21–105)	52.4 (21–105)	54.8 (25–103)	0.732[a]
Female: *n* (%)	34 (39.1%)	28 (38.8%)	6 (40.0%)	1.000[b]
Male: *n* (%)	53 (60.9%)	44 (61.1%)	9 (60.0%)	

[a]Student's *T*-test, [b]Fisher's Exact Test.

Supplementary Table 10. The parameters used to construct the four prediction models, based on the initial five-fold CV results.

Lasso (22 probe sets)	EN (252 probe sets)	XGBoost (269 probe sets)	LightGBM (404 probe sets)
Alpha = 1	Alpha = 0.05	Learning rate = 0.1	Learning rate = 0.05
Lambda = 4.335	Lambda = 45.205	Gamma = 0.425	Max. tree depth = 5
		Max. tree depth = 3	Num. of leaves = 30
		Min. child weight = 9.5	Min. data in a leaf = 22
		Subsample ratio = 1	Min. sum hessian in a leaf = 0.001
		Column sample ratio = 0.8	Subsample ratio = 0.85
		L1 regularization = 0	Column sample ratio = 0.6
		L2 regularization = 3.5	L1 regularization = 0.4
		Num. boosting rounds = 366	L2 regularization = 0.4
			Min. gain to split = 0
			Num. boosting rounds = 970
